# Optimization of Glucose Dehydrogenase Immobilization Strategies in a 3D-Printed Millireactor

**DOI:** 10.3390/mi15121514

**Published:** 2024-12-20

**Authors:** Vilim Marijan Boroša, Kristian Koštan, Renata Vičević, Ivan Karlo Cingesar, Domagoj Vrsaljko, Bruno Zelić, Ana Jurinjak Tušek, Anita Šalić

**Affiliations:** 1 University of Zagreb Faculty of Chemical Engineering and Technology, Marulićev Trg 19, HR-10000 Zagreb, Croatia; vborosa@fkit.hr (V.M.B.); kkostan@fkit.hr (K.K.); rvicevic@fkit.unizg.hr (R.V.); icingesar@fkit.unizg.hr (I.K.C.); dvrsal@fkit.unizg.hr (D.V.); asalic@fkit.unizg.hr (A.Š.); 2 Department of Packaging, Recycling and Environmental Protection, University North, Trg dr. Žarka Dolinara 1, HR-48000 Koprivnica, Croatia; 3 University of Zagreb Faculty of Food Technology and Biotechnology , Pierottijeva Ulica 6, HR-10000 Zagreb, Croatia; ana.tusek.jurinjak@pbf.unizg.hr

**Keywords:** enzyme immobilization, 3D-printed millireactor, glucose dehydrogenase, alginate gel, enzyme stability and activity

## Abstract

Enzymatic reactions play an important role in numerous industrial processes, e.g., in food production, pharmaceuticals and the production of biofuels. However, a major challenge when using enzymes in industrial applications is maintaining their stability and activity, especially under harsh operating conditions. To solve this problem, enzyme immobilization techniques have been developed. Immobilization involves fixing the enzymes on solid supports, which increases their stability, enables their reusability and facilitates the easy separation of reaction mixtures. In addition, immobilized enzymes are ideal for continuous flow systems such as millireactors, where they allow better control of reaction conditions, improving efficiency and product consistency. Glucose dehydrogenase is an important enzyme in biotechnology, particularly in biosensors and the production of biofuels, as it catalyzes the oxidation of glucose to gluconolactone, reducing NAD^+^ to NADH. However, like many other enzymes, it tends to lose activity over time. The immobilization of glucose dehydrogenase in a millireactor provides a controlled environment that increases the stability and activity of the enzyme. The aim of this study was to investigate the effects of different immobilization strategies on the performance of glucose dehydrogenase in a 3D printed millireactor. The enzyme was immobilized in alginate gel in three immobilization strategies: as beads, on the bottom surface, and on both the top and bottom surfaces of the millireactor. The results showed that the application of the enzyme on both surfaces improved the glucose conversion two-fold compared to immobilization in beads and four-fold compared to immobilization only on the bottom surface. The dual-surface enzyme immobilization strategy showed the highest efficiency, achieving the highest conversion of 95.76 ± 1.01% (*τ* = 131 min) and NADH productivity of 0.166 ± 0.01 mmol/(L·min) (*τ* = 7.11 min) combined with operational stability over five days. Effective diffusion rates comparable to those of aqueous solutions confirmed the suitability of alginate gels for biocatalysis. These advancements highlight the potential of this modular and scalable platform for various biotechnological applications.

## 1. Introduction

Enzymes have numerous applications in various fields such as food production, fine chemistry, the textile industry, molecular biology, pharmacology, biofuels, etc., with an increasing number of applications in new fields [1,2,3,4,5,6]. They are an essential part of industrial biotechnology as they offer numerous advantages over chemical catalysts: high catalytic activity, high specificity for substrates and solubility in organic solvents. In addition, enzymes lead to energy savings and a lower environmental impact as they allow reactions to occur under mild conditions such as room temperature and atmospheric pressure, unlike conventional chemical catalysts which usually require high temperatures and pressures [7,8].

However, enzymes also have some serious disadvantages: they are expensive, very sensitive to denaturants [9] and, most importantly, they lose activity and stability over time [10]. Another important property of enzymes, stability, refers to the conditions such as temperature, pH, presence of co-solvents, etc., under which enzymes retain their structure and activity [11].

To overcome these problems, enzymes are immobilized by one of the selected methods [6,12,13,14,15], which minimizes the negative effects on enzyme activity and maximizes the efficiency of enzymatic reaction, including minimizing the cost, improving the catalytic activity, easy separation and protecting the enzyme from degradation or inactivation, etc. [16]. In enzyme immobilization, enzymes are chemically or psychically bound to a solid surface while retaining their biological activity and tertiary structure [7,12]. To achieve this, the functional groups in the active site of the enzyme should not be involved in the immobilization process [7]. As a result, immobilized enzymes enable the continuous and repeated performance of chemical reactions [7,17]. These immobilized enzymes have several advantages over conventional methods that use resuspended enzymes. These include the fact that they are insoluble and can be regenerated for repeated use in reactors, allow easier control of the reactor system, and give the enzymes higher thermal and pH stability [7]. For all these reasons, immobilization extends the life of the enzyme and makes the process more cost-effective and environmentally friendly. Enzymes can be physically (adsorption and entrapment) and chemically (cross-linking and carrier binding) bound to the solid support [7], and the method chosen depends on the type of the enzyme and solid support as well as the application of the enzyme [18].

Both chemical immobilization and physical binding have some disadvantages that can affect the efficiency of the enzymatic reaction, such as possible changes in the structure of the enzymes in chemical immobilization and the risk of leakage of the enzyme in physical binding due to weak bonds [7]. An interesting method to overcome these problems is the enzyme entrapment method, in which the enzyme is protected by gel entrapment and microencapsulation, i.e., by forming the polymer (gelatine, alginate, polyacrylamide) in the presence of an enzyme solution. The disadvantage of this method is the possible impairment of the physical, chemical and kinetic properties of the immobilized enzyme [7,19].

Millireactors are increasingly being used to further optimize and intensify the processes [20]. These reactors with a reduced volume, a channel length, a hydraulic diameter [21] and typical internal dimensions from 1 to 10 mm [22] have many advantages compared to conventional reactors. Millireactors offer the precise control of the conditions in the reactor, efficient mixing, lower reagent and catalyst requirements, a high surface area to volume ratio resulting in improved heat and mass transfer and operational safety making them very effective for process intensification [20,23]. With laminar flow and a short residence time, millireactors enable faster chemical reactions with higher yields, making them ideal for process intensification and highly efficient for industrial applications [23].

Three-dimensional printing has significantly advanced the development of millireactors for chemical applications and offers several advantages, such as fast design, low cost and the ability to use a wide range of materials. This technology makes it possible to explore new possibilities and optimize millireactors down to the smallest detail and promote their dissemination in many fields [24], such as protein detection, cell deposition, bacterial community studies, tissue engineering, organ-on-a-chip models, etc. [25]. Millireactors have been 3D-printed using five technologies: stereolithography, fused deposition modelling, multi-jet modelling, selective laser sintering and selective laser melting. However, each of these techniques brings with it specific technical and material challenges, i.e., problems in printing small, complex geometry parts, the precision of 3D printers, particularly when fabricating two-part systems that must fit together seamlessly, knowledge about material selection, etc. that need to be thoroughly addressed before 3D printing [26].

This study focuses on maximizing the performance of the enzyme, glucose dehydrogenase, in 3D-printed millireactors using different alginate gel immobilization strategies. In particular, a novel geometry of the millireactor is presented that eliminates dead zones and minimizes leakage, common in 3D-printed millireactors. The study also investigates a simple but effective approach for assembling the reactor that ensures practicality and reliability. In addition, different enzyme immobilization strategies were investigated to optimize the performance of the biocatalytic system, providing valuable insights to improve the efficiency and applicability of such reactors. In a first step, a new millireactor design was proposed based on the results of computational fluid dynamics (CFD) simulations. The enzyme was immobilized in alginate gel using three immobilization strategies: as beads, on the bottom surface, and on both the top and bottom surfaces of the millireactor. After immobilization of the glucose dehydrogenase, glucose oxidation reactions were performed to evaluate the efficiency of each system. Glucose oxidation was monitored by decreasing the glucose concentration, while the formation of NADH over time was an indicator of enzymatic activity.

## 2. Materials and Methods

### 2.1. Materials

#### Chemicals

The enzyme, glucose dehydrogenase (GDH), from *Pseudomonas* sp. (1.1.1.47, CAS Number: 9028-53-9, S.A. = 200 U/mg), bovine serum albumin (BSA), nicotinamide adenine dinucleotide (NAD^+^), reduced nicotinamide adenine dinucleotide (NADH) and Tris (hydroxymethyl) aminomethane (TRIS) were purchased from Sigma Aldrich (Vienna, Austria). The glucose PAP test was purchased from Dijagnostika d.o.o. (Zagreb, Croatia). Glucose and sodium alginate were purchased from Fisher Scientific (Loughborough, UK). Hydrochloric acid (HCl), calcium chloride anhydrous and isopropyl alcohol were purchased from Gram-Mol d.o.o. (Zagreb, Croatia). Sodium phosphate monohydrate and sodium phosphate pentahydrate were purchased from Kemika (Zagreb, Croatia). Anycubic Standard Clear resin was purchased form Anycubic Technology Co., Ltd. (Shenzhen, China).

All the chemicals were of analytical grade and were used without further purification unless otherwise stated. Solutions were prepared with ultrapure water, and all the reagents were stored according to the manufacturer’s recommendations.

### 2.2. Methods

#### 2.2.1. Glucose Dehydrogenase Assay

The GDH activity was determined using the glucose oxidation reaction, in which NADH and glucono-*δ*-lactone are formed. The concentration of NADH formed is proportional to the glucose concentration; therefore, the rate of NADH formation correlates with the reaction rate and consequently with the activity of the GDH. The reaction mixture contained 700 µL of 20 mmol/L TRIS-HCl buffer pH 7, 100 µL of 0.2 mol/L glucose solution and 100 µL of 0.022 mmol/L NAD^+^. The quartz cuvette with the prepared mixture was thermostatted in a water bath at *T* = 40 °C for 10 min (Thermomix 1420, Braun, Hamburg, Germany). The reaction was started by adding the GDH from *Pseudomonas* sp. (100 µL) to the mixture. The change in absorbance over 60 s was monitored spectrophotometrically (UV-1601, Shimadzu, Tokyo, Japan) at a wavelength of *λ* = 340 nm. One enzyme unit was defined as the amount of enzyme required to oxidize 1 µmol of glucose per minute at pH 7 and *T* = 40 °C to glucono-*δ*-lactone in the presence of NAD^+^.

#### 2.2.2. Determination of the Enzyme Concentration Using the Linearized Bradford Assay

The concentration of the GDH from *Pseudomonas* sp. was determined using the linearized Bradford assay [27]. First, a BSA solution with a concentration of 1 mg/L was prepared, which corresponds to an absorbance of 0.66 at *λ* = 280 nm in a quartz cuvette. This solution was then diluted to obtain a concentration range of 0 to 100 mg/L. In a plastic cuvette, 500 µL of the Bradford reagent and 500 µL of the sample were added. The mixture was homogenized briefly. After 5 min, the absorbance of the sample was measured with a UV–VIS spectrophotometer at wavelengths of *λ* = 595 nm and *λ* = 450 nm. A calibration curve was created based on the measured values and known concentrations. A linear relationship between absorbance and protein concentration was determined for concentrations from 0 to 50 mg/L. The calibration curve was used to determine the unknown concentration of GDH in the samples. Each measurement was performed in triplicate, and the results are expressed as average ± standard deviation (st.dev.).

#### 2.2.3. Determination of Glucose Concentration Using the Enzymatic GOD-PAP Method

The glucose concentration in the samples was determined using the enzymatic GOD-PAP method, in which the enzyme, glucose oxidase, catalyzes the oxidation of glucose with the release of hydrogen peroxide. In the next step, the enzyme reacts with 4-hydroxybenzoate and 4-aminoantipyrine in the presence of peroxidase and forms a red quinoneimine dye. The intensity of the dye is proportional to the amount of glucose present in the sample. To create the calibration curve, a glucose standard solution with a concentration of 5.55 mmol/L was diluted into six solutions with concentrations between 0.17 and 5.55 mmol/L. The measurement was performed by adding 10 μL of the sample and 1 mL of the working reagent (a mixture of R1: 4-aminoantipyrine, glucose oxidase, peroxidase and R2: phosphate buffer pH 7.5, phenol, detergent and stabilizer) to empty Eppendorf tubes. The mixture was homogenized and incubated at *T* = 25 °C for 20 min. After exactly 20 min, the absorbance was measured spectrophotometrically at a wavelength of *λ* = 500 nm. A blank sample was prepared in the same way, using 10 μL of ultrapure water instead of the sample. The resulting calibration curve represents the dependence of the absorbance on the known glucose concentration. Each measurement was performed in triplicate, and the results are expressed as average ± standard deviation (st.dev.).

#### 2.2.4. Spectrophotometric Measurement of the NADH Concentration

The NADH concentration in the samples was determined by measuring the absorbance at *λ* = 340 nm. Firstly, solutions with different NADH concentrations, ranging from 0.018 to 0.300 mol/L were prepared. At the wavelength *λ* = 340 nm, which corresponds to the absorption maximum of NADH, the absorbance of the samples was measured in quartz cuvettes. A calibration curve was created on the basis of the absorbance values obtained and known concentrations. The unknown concentrations of NADH in the samples were determined using the calibration curve. Each measurement was performed in triplicate, and the results are expressed as average ± standard deviation (st.dev.).

#### 2.2.5. Millireactor Design and Fabrication

A commercially available photocurable resin was used for the 3D printing of the millireactor. This resin is a formulation that contains a photoinitiator along with acrylate and epoxy compounds. The Anycubic Standard Clear resin (Anycubic Technology Co., Ltd., Shenzhen, China) contains ethylene oxide-propylene oxide block copolymer dimethacrylate (30–40%), polyethylene glycol diacrylate (10–25%), tripropylene glycol diacrylate (15–25%), poly(oxy-1,2-ethanediyl)-ethoxylated bisphenol A diacrylate (10–20%) and bis(2,4,6-trimethylbenzoyl)phenylphosphine oxide (2–5%). Isopropyl alcohol was used to remove residual uncured resin from the 3D-printed structures.

**Figure 1:**
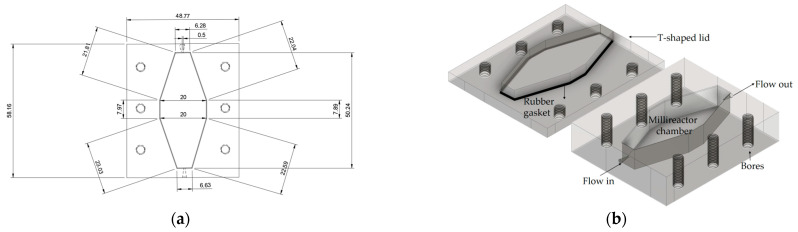
Schematic representation of (**a**) design specifications (in mm) with height = 5 mm and average hydraulic diameter = 6.69 ± 0.98 mm and (**b**) key millireactor elements.

The millireactor (Figure 1) was designed using Autodesk Fusion CAD software v2.0.20256 (Autodesk, Inc., San Francisco, CA, USA). The models were prepared for 3D printing using Chitubox Slicing Software v1.9.4 (Shenzhen CBD Technology Co., Ltd., Shenzhen, China). A Digital Light Processing (DLP) 3D printer, Anycubic Photon M3 (Anycubic Technology Co., Ltd., Shenzhen, China), was used to produce the reactors. The layer height, which corresponds to the z-axis resolution, was set to 50 μm. During the entire 3D printing process, the temperature of the resin in the vat was kept at room temperature (*T* = 25 °C). The dimensions of the printed millireactor differed from those of the theoretical model by 0.97 ± 0.71%.

#### 2.2.6. GDH Immobilization in an Alginate Gel

**Figure 2:**
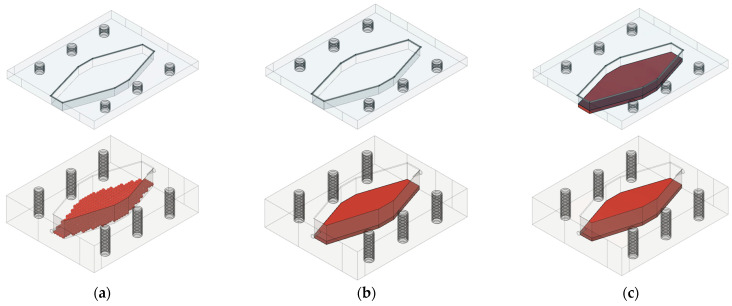
Different GDH immobilization strategies (**a**) as beads, (**b**) on the bottom surface of the millireactor, and (**c**) on both the bottom and the top surface of the millireactor.

For the preparation of the 2% (*w*/*v*) alginate gel, sodium alginate was dissolved in ultrapure water by mixing at *T* = 60 °C. After cooling to room temperature (*T* = 25 °C), GDH (*γ* = 10 mg/L) was added to 1 mL of the solution. The solution was mixed for a further 15 min to obtain a homogeneous solution. Separately, a cross-linking solution, 2% (*w*/*v*) CaCl_2_, was prepared in ultrapure water. According to the first immobilization strategy (Figure 2a), the alginate gel beads were formed by dropping the alginate solution into the stirred CaCl_2_ solution (*V* = 100 mL) from a height of about 20 cm using a syringe and a needle at room temperature. The beads were allowed to solidify in the calcium solution for 1 h. In the second strategy, the alginate solution with the enzyme (1 mL) was poured onto the bottom surface of the millireactor to form the alginate gel (Figure 2b). In the third strategy, the alginate solution with the enzyme was poured onto the bottom surface (0.5 mL) and the top surface (0.5 mL) of the millireactor (Figure 2c). For the second and third strategy of immobilization, CaCl_2_ was sprayed over the surface and was allowed to solidify for 1 h after the excess CaCl_2_ was removed via rinsing.

#### 2.2.7. Glucose Oxidation in a Millireactor

**Figure 3:**
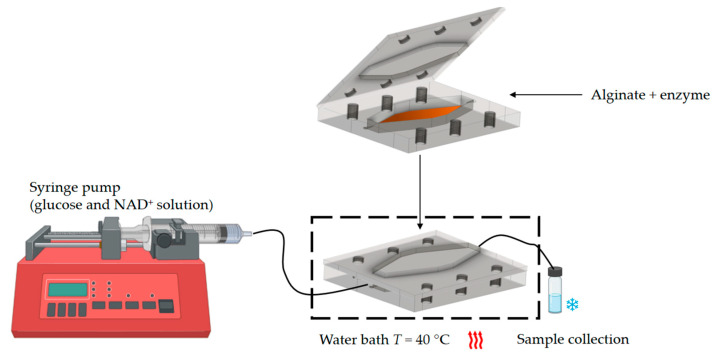
Experimental setup used for glucose oxidation.

To perform glucose oxidation in a millireactor (Figure 3), a 5 mmol/L equimolar solution of glucose and NAD^+^ was prepared in 20 mmol/L TRIS-HCl buffer at pH 7. The prepared solution was added to a stainless steel high-pressure syringe (8 mL, Harvard Apparatus, Holliston, MA, USA). The syringe was placed on the pump (PHD 4400 Syringe Pump Series, Harvard Apparatus, Holliston, MA, USA) and connected with PTFE tube to a millireactor. The millireactor was submerged in a water bath with a heat regulation system (Thermomix 1420, Braun, Hamburg, Germany). In all the experiments, the flow rate was varied from 25 to 400 µL/min to investigate the influence of the residence time on glucose oxidation. The output stream was collected in a vial placed on ice to stop the reaction. After collecting 500 µL of a sample, the sample was additionally filtered (Filter Chromafil Xtra PTFE-20/25; 0.2 µm, 25 mm, Macherey-Nagel GmbH CoKG, Düren, Germany). The glucose and NADH concentrations were measured in all the collected samples.

#### 2.2.8. Operational Stability

The operational stability of the system was evaluated by performing a continuous biotransformation over five days at *T* = 40 °C. The experiment was performed as described in Section 2.2.7. at a constant flow rate of 25 µL/min. Samples were collected daily, and the glucose concentration was determined as described.

#### 2.2.9. Diffusion Measurement of Glucose and NADH Through Alginate Gel

**Figure 4:**
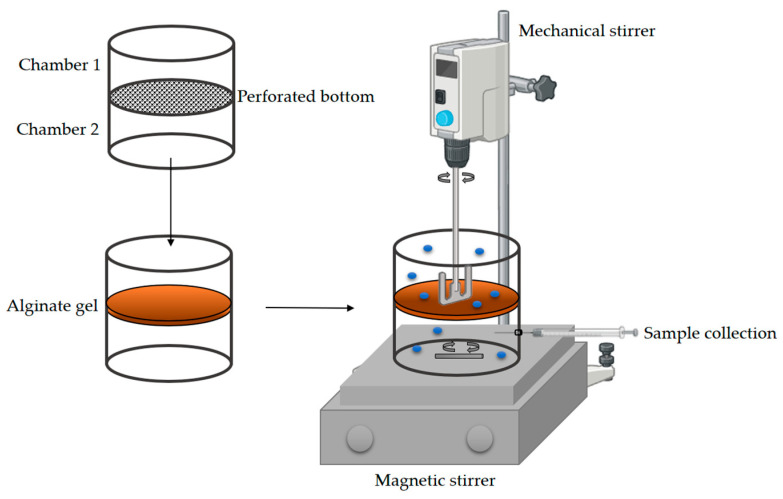
Experimental setup for measuring the diffusion of glucose and NADH through alginate pores.

The diffusion of glucose and NADH molecules through the pores of the alginate gel was measured using a custom-made cell with two chambers with the specific dimensions *V* = 180 mL, *d* = 8.5 mm. The chambers were separated by a perforated bottom to which alginate gel was applied (*A* = 56.74 cm^2^, *h* = 1.7 mm, Figure 4). A glucose (5 mmol/L) or NADH (1 mmol/L) solution was added to the upper chamber, while ultrapure water was added to the lower chamber. Both solutions were constantly stirred to allow the molecules to diffuse through the hydrogel from one solution to the other due to the concentration gradient between the two reservoirs. At specific time intervals, every 10 s, a sample was taken from both chambers, and the glucose or NADH concentration was measured.

To determine the effective diffusivity coefficient (*D_eff_*) through the alginate gel, the generalized and linearized derivation of Fick’s second diffusion law according to Equations (1) and (2) was used [28]:

(1) $$D_{eff}=\frac{1}{\beta \cdot t}\ln\left(\frac{c_{1}(t)-c_{2}(t)}{c_{1,0}-c_{2,0}}\right)$$

with

(2) $$\beta =\left(\frac{A_{H}}{W_{H}}\right)\left(\frac{1}{V_{1}}+\frac{1}{V_{2}}\right)\;$$

where *c*_1,0_ is the initial concentration of glucose or NADH in chamber 1, *c*_2,0_ is the initial concentration of glucose or NADH in chamber 2. *c*_1_(*t*) and *c*_2_(*t*) represent the concentration of glucose or NADH in chamber 1 and chamber 2 after time *t*. *A*_H_ is the effective cross-sectional area of diffusion, *W*_H_ is the thickness of the alginate gel and *V*_1_ and *V*_2_ are the volumes of chambers 1 and 2, respectively.

#### 2.2.10. Computational Fluid Dynamics (CFD) Modelling

The models designed in Autodesk Fusion were exported in the dxf format and imported into COMSOL Muliphysics 5.6 (COMSOL Inc., Stockholm, Sweden). The software was used to solve partial differential equations to obtain a 2D velocity model of a single-phase laminar flow in a millireactor. COMSOL Multiphysics uses the finite element method, coupled with adaptive meshing and error control, using a range of numerical solvers. The default meshing typically divides the geometry into triangular elements, but users can modify the mesh to different shapes and sizes as required [29]. For this study, the model was developed under room temperature conditions using the Navier–Stokes equations for incompressible fluids along with the continuity equation to simulate a constant flow (400 µL/min). Standard boundary conditions were applied, including no-slip conditions at the walls, fully developed laminar flow, specified inflow velocity and zero relative pressure at the outflow. One of the default COMSOL Multiphysics 5.6 options was chosen for the meshing: a physically controlled finer triangular mesh. This mesh consisted of 48,138 domain elements and 4850 boundary elements, as the initial results with a normal mesh with 30,804 domain elements and 3877 boundary elements led to less accurate results. Additionally, extra fine and extremely fine meshes were tested, but the results showed no significant improvement compared to the finer mesh that was finally chosen.

## 3. Results

### 3.1. Millireactor Design

Millireactors have attracted considerable attention in various fields such as chemical synthesis, pharmaceutical development and environmental applications due to their improved reaction control, enhanced heat and mass transfer and reduced reagent consumption. A particularly promising area of research is the use of immobilized enzymes in millireactors, which are often embedded in hydrogels or alginate to enhance their stability and activity [30]. These enzyme-immobilized systems are becoming increasingly important in millireactor applications as they enable continuous processes with high catalytic efficiency, improved reusability of the biocatalysts and minimal contamination of the product stream. Traditionally, rectangular millireactors with resealable lids [30] have been used in combination with alginate hydrogels due to their simple design and compatibility with conventional manufacturing techniques. However, a major limitation of these designs is the presence of significant dead volumes—areas within the reactor where the liquid flow is minimal or stagnant. These dead volumes result in inefficient mixing, lower reaction efficiency and an uneven residence time distribution, undermining the potential benefits of millireactor technology. In addition to the problem of dead volume, many of the millireactors presented so far have a flat reactor cover, which leads to the leakage of the reaction mixture during operation. This leakage poses a major challenge, especially when it comes to maintaining the integrity of the reaction process and ensuring consistent results.

In this study, a novel 3D-printed millireactor design was proposed to solve the aforementioned problems, minimize dead volume and prevent leakage. As shown in Figure 1b, a simple millireactor design consisting of a chamber, a T-shaped lid, a rubber gasket, and an inlet and outlet was proposed. The design deviates from the conventional rectangular geometry to reduce dead zones. The total volume of the millireactor was 3554.4 µL, with the characteristic dimensions shown in Figure 1b. In addition, a T-shaped lid configuration was proposed to avoid leakage and reduce it to a negligible level. Six holes were added to secure the lid to a main chamber with screws. A similar lid configuration was also recently proposed by Bajić et al. [31]. This configuration provides a tighter seal between the reactor components and significantly improves the operational safety compared to the conventional flat lids. Furthermore, in this design, the inlet and outlet were positioned on the side of the millireactor, avoiding contact with the lid. This allowed the lid to be easily moved without disturbing the rest of the millireactor, and the millireactor could be operated in both closed and open modes.

**Figure 5:**
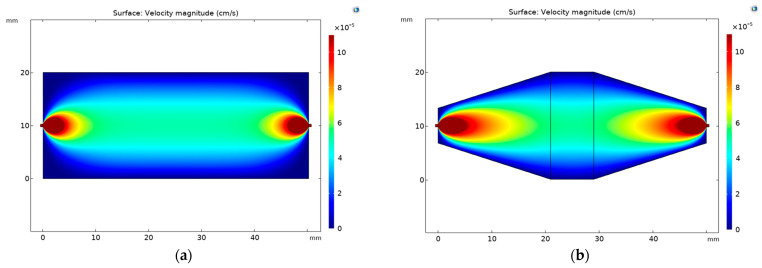
Fluid flow characteristics in (**a**) a rectangular millireactor and (**b**) a millireactor proposed in this research for a flow rate of 400 µL/min.

To determine the optimal design, computational fluid dynamics (CFD) simulations were performed to evaluate the flow within the proposed millireactor design and compare the results with those of a conventional rectangular millireactor. For the simulations, the properties of pure water at *T* = 20 °C were used as the working fluid. The flow was characterized as laminar, and the system was assumed to be isothermal and in a steady state. As shown in Figure 5, the highest liquid velocities were observed in the centre of the reactor in both millireactor configurations, which is consistent with the results of Bajić et al. [31] and Macown et al. [32]. However, the range of high velocity was larger in the proposed design.

When comparing the dead volumes where the flow of reactants or products is stagnant or minimal, resulting in inefficient mixing, incomplete reactions and prolonged residence times, the CFD models (Figure 5) show significantly larger stagnant zones in the rectangular millireactor (Figure 5a). To reduce the formation of these dead volumes, the reactor design was modified, as shown in Figure 5b, by cutting off the corners to reduce the stagnant areas and improve the flow dynamics within the reactor.

### 3.2. Glucose Oxidation in a 3D-Printed Millireactor

Before performing the glucose oxidation reaction with GDH from *Pseudomonas* sp., three strategies were used to immobilize the enzyme: (a) in alginate beads, (b) in the form of an alginate hydrogel directly on the bottom surface of the millireactor and (c) in the form of an alginate hydrogel on both the bottom and top surfaces of the millireactor. In each strategy, the effect of immobilization on the enzyme activity was investigated, focusing on the available surface area for enzyme–substrate interactions. In order to compare the results, it was important to ensure that the enzyme concentration was the same in all three immobilization strategies.

In the first strategy, GDH was encapsulated in alginate beads. Alginate is a biopolymer derived from brown algae that is commonly used for enzyme immobilization due to its biocompatibility, ease of gelling and ability to form a stable matrix under mild conditions [33]. Although this strategy offers several advantages, such as the protection of enzyme activity, porous structure and reusability, it also has its limitations [34]. Although alginate beads provide a protective environment for the enzyme and enable reusability, they also lead to mass transfer limitations upon diffusion. The substrate must diffuse into the bead matrix, which can slow down the reaction kinetics. In addition, the total surface area available for interaction between the enzyme and substrate is reduced compared to surface-bound systems, potentially leading to lower overall reaction rates [33,34]. During immobilization, 33 alginate beads were formed, some in the shape of a sphere (diameter_average_ = 3 mm) and the others in the shape of a cylinder (diameter_average_ = 4 mm, length_average_ = 1 mm).

In the second strategy, GDH was directly immobilized on the bottom surface of the millireactor in the form of an alginate hydrogel with a thickness of 1.4 mm. In hydrogel immobilization, a soft, hydrated polymer network is formed in which the enzyme is trapped while the substrate can diffuse through the gel matrix. This strategy creates a solid interface where interactions between the enzyme and substrate occur, and the hydrogel provides stability and a structured environment for the enzyme [30]. By immobilizing the enzyme only on the bottom surface, the available surface area for the reaction is limited to one level of the millireactor. Accessibility is improved compared to immobilization with beads because the hydrogel layer is usually thinner and has a more direct interface with the flowing substrate solution. This minimizes the length of the diffusion path for the substrate to reach the enzyme and reduces the diffusion limitations compared to larger alginate beads. In addition, the flat geometry of the hydrogel provides a more uniform exposure of the enzyme to the substrate stream which improves the reaction efficiency despite the alginate matrix. While this strategy improves the accessibility of the enzyme to the substrate compared to immobilization with beads, it still limits the total surface area available for the reaction, which could limit the reaction rate compared to more extensive immobilization strategies.

**Figure 6:**
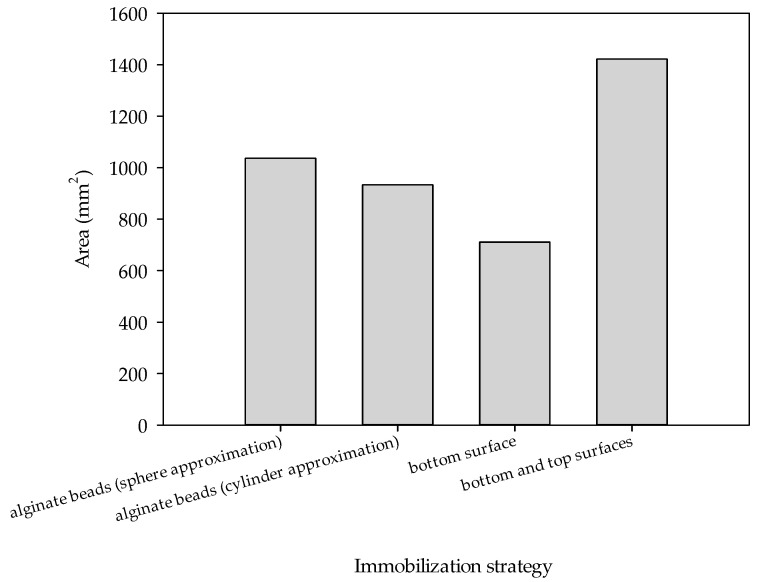
Comparison of the available surface area depending on the immobilization strategy.

In the third strategy, GDH was immobilized in the form of an alginate hydrogel on both the bottom and top surface of the millireactor with a thickness of 0.7 mm on each side. This approach significantly increased the available surface area, reduced the thickness of the gel and improved the enzyme–substrate interactions (Figure 6). By distributing the enzyme over two surfaces, the system maximized the exposure of GDH to the substrate, allowing for more efficient diffusion and higher reaction rates. The increased surface area in this approach provided more sites for the enzyme to interact with the substrate, which improved the overall catalytic activity. The double surface immobilization also minimized dead volumes—areas where reactant flux is stagnant or minimal—optimizing the substrate flux and reducing the diffusion limitations.

The immobilization of the GDH for glucose oxidation reactions was performed to evaluate the efficiency of each system. It should be noted that the enzyme activity in solution was 180 ± 12 U/mg. The immobilization in alginate was assumed not to affect the enzyme activity, and at an enzyme concentration of approximately 10 mg/L in all the experiments, the specific activity (the ratio of volumetric activity to enzyme contraction) was calculated to be 18 ± 1.2 U/mg. During the process, the change in glucose and NADH concentration as well as the enzyme concentration at the reactor outlet were monitored. No release or “leakage” of the enzyme from the alginate gel was observed.

**Figure 7:**
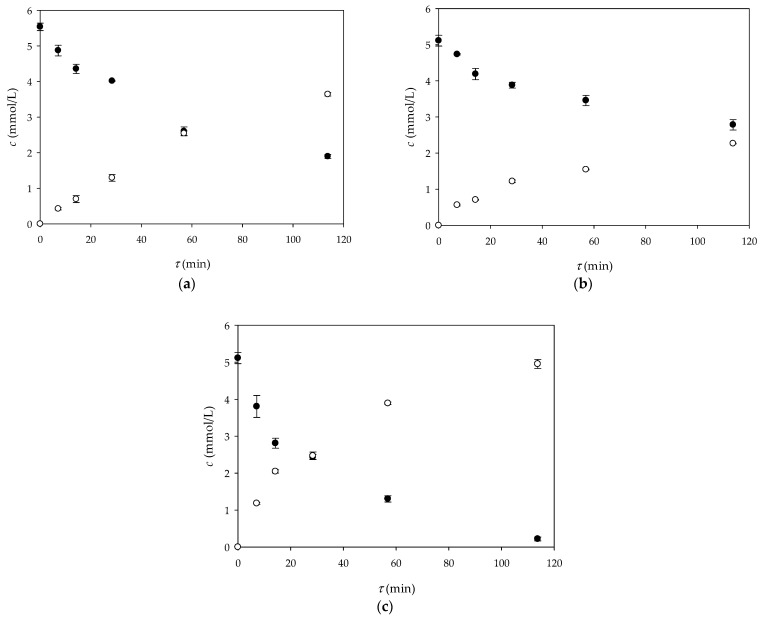
Influence of residence time on the concentration of glucose (•) and NADH (◦) when the enzyme was immobilized in (**a**) alginate beads and alginate hydrogen, (**b**) bottom surface, and (**c**) bottom and top surface on the millireactor.

As can be seen from Figure 7, the formation of NADH is directly proportional to the oxidation of glucose. In all the immobilization strategies, longer residence times led to a higher substrate conversion, as the reactants had more time to interact with the immobilized enzyme. However, significant differences in the reaction rate and overall efficiency were observed depending on the enzyme immobilization strategy.

The fastest and most efficient glucose oxidation observed in the system in which GDH was immobilized on both the lower and upper surfaces of the millireactor (Figure 6) can be attributed to the coupled effects of increasing the surface-to-volume ratio and decreasing the diffusion length. The immobilization of the enzyme on both surfaces significantly increases the total surface area available for enzyme–substrate interactions, which directly increases the reaction rate. The larger surface area allows more active sites of the enzyme to be exposed to the substrate, resulting in a greater number of enzyme–substrate interactions per unit time. In addition, the increased surface area also reduces the diffusion length, i.e., the distance over which the substrate molecules have to diffuse in order to reach the enzyme. A shorter diffusion distance increases the rate at which substrate molecules can be converted into products, as they can reach the enzyme faster and with less delay. This reduction in diffusion restriction combined with the increased surface area for enzyme binding accelerates the reaction kinetics. The result is a faster decrease in glucose concentration and a faster formation of NADH, highlighting the importance of both surface area-to-volume ratio and diffusion length in optimizing biocatalytic reactions.

**Figure 8:**
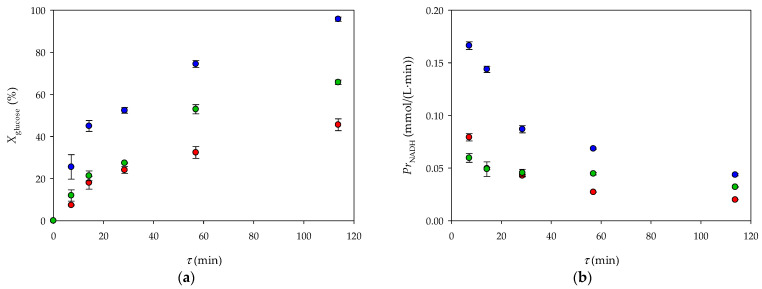
Change in (**a**) conversion and (**b**) productivity upon immobilization of the enzyme in alginate beads (•) and alginate hydrogen, bottom surface (•), and bottom and top surface of the millireactor (•).

The influence of the immobilization strategy is perhaps most evident when comparing the conversion and productivity of the three strategies (Figure 8). Conversion and productivity were highest in the millireactor in which the enzyme was immobilized in two layers (both on the bottom and on the top surface). The comparison illustrates the advantage of maximizing the enzyme availability and distribution within the millireactor to achieve better reaction outcomes.

**Figure 9:**
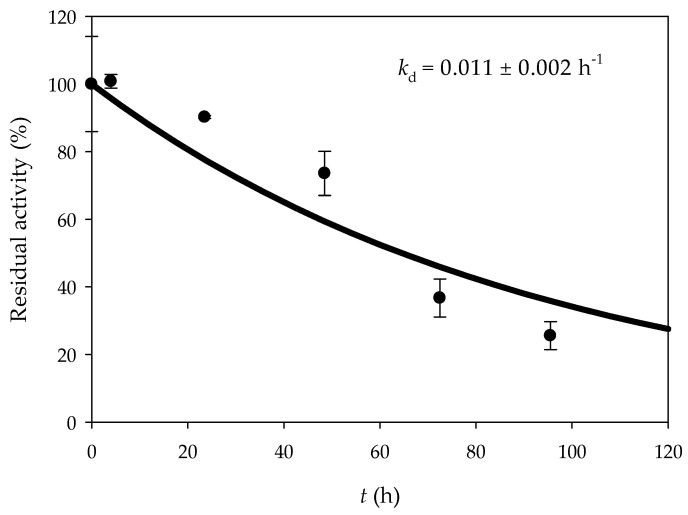
Enzyme deactivation.

Another critical aspect of experiments with immobilized biocatalysts is their reusability. To investigate the potential for prolonged reuse, experiments were performed with alginate hydrogel. After each initial experiment, the millireactor was washed with 20 mmol/L TRIS-HCl buffer at pH 7, and the operational stability of the system was evaluated via a continuous biotransformation for five days at *T* = 40 °C with a constant flow rate of 25 µL/min. Samples were taken daily, and the glycerol concentration was determined. As shown in Figure 9, a decay rate constant (*k*_d_) of 0.011 ± 0.002 h^−1^ was determined. According to the model predictions, the system can maintain activity for almost five days.

### 3.3. Diffusion of Glucose and NADH Through Alginate Gel

The effective diffusivity of molecules through alginate gel is a critical factor affecting the performance of enzyme immobilization systems, particularly in biotechnological applications [35]. The alginate gel structure is highly porous, which can allow the diffusion of small molecules, but the extent of diffusion depends on both the network properties of the gel and the molecular properties of the solutes [36]. Due to the restricted movement of molecules through the polymeric matrix of the gel, the effective diffusivity is generally reduced compared to free diffusion in aqueous solutions. Factors such as the gel concentration, the cross-linking density and the pore size distribution directly have a direct effect on the diffusion rate [37]. Higher alginate concentrations or stronger cross-linking lead to smaller pores, which can slow down the diffusion of larger molecules, while lower concentrations increase the diffusivity but can reduce the mechanical stability of the gel. In addition, effective diffusivity can also be influenced by the environmental conditions such as temperature, pH and the presence of other solutes [38]. In enzyme immobilization systems, the optimization of the effective diffusivity is crucial for ensuring efficient substrate conversion rates and maintaining the enzyme activity over an extended period of time in continuous-flow bioreactors.

**Figure 10:**
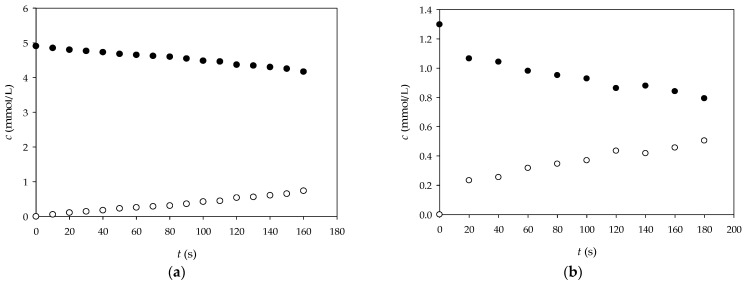
Changes in (**a**) glucose and (**b**) NADH concentrations in chamber 1 (•) and chamber 2 (◦) during the time.

**Figure 11:**
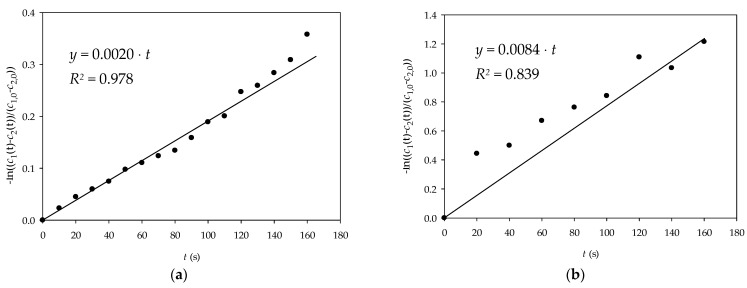
Estimation of the effective diffusivity coefficient for (**a**) glucose and (**b**) NADH.

In this work, the effective diffusivity coefficients of glucose and NADH through alginate gel were estimated based on the experimental data. The changes in the concentrations of glucose and NADH in both chambers during the experiment are shown in Figure 10. As expected, the concentrations of the analyzed components in chamber 1 decreased with time, while the concentrations in chamber 2 increased accordingly. By rearranging Equation (1), a linear relationship between $-ln\left(\frac{c_{1}\left(t\right)-c_{2}\left(t\right)}{c_{1,0}-c_{2,0}}\right)\;$ and *t* was obtained (Figure 11), and the effective diffusivity of the analyzed components was calculated based on the slope of the obtained lines and the information on the effective surface area and thickness of the alginate gel. The results obtained showed that the effective diffusivity of glucose through the alginate film was *D_eff_*_,GLC_ = 5.396 × 10^−8^ m^2^/s and for the NADH *D_eff_*_,NADH_ = 2.267 × 10^−9^ m^2^/s, and the results obtained were in the same range as the effective diffusivity of the analyzed components in the aqueous solution (*D_eff_*_,GLCaq_ = 1.482 × 10^−8^ m^2^/s and for the NADH *D_eff_*_,NADHaq_ = 8.268 × 10^−9^ m^2^/s), which means that the alginate gel structure allows the diffusion of the reaction mixture components, which is crucial for an effective biotransformation process.

## 4. Conclusions

This study presents the design and evaluation of a novel 3D-printed millireactor tailored to address the critical problems of conventional rectangular millireactors, including dead volume and leakage. The proposed millireactor design with a T-shaped lid and an optimized inlet and outlet arrangement minimizes dead zones and increases the operational safety by reducing leakage. CFD simulations confirmed improved flow dynamics and showed a reduction in stagnant areas compared to the conventional designs. It should also be noted here that the comparison between the design proposed in this study and the traditional rectangular millireactor is limited solely by the issue of dead volume and leakage; no quantitative performance comparison, either experimental or numerical, was performed. Future work should focus on numerical validation and experimental analysis to further confirm and optimize the performance of the new design.

The immobilization of enzymes was investigated using three approaches: encapsulation in alginate beads, alginate hydrogel immobilization on the bottom surface and alginate hydrogel immobilization on both the bottom and top surface of the millireactor. The results showed that dual-surface enzyme immobilization significantly improved glucose conversion, with rates twice as high as the immobilization in the alginate beads and four times as high as the immobilization on the bottom surface only. This strategy exhibited the highest efficiency, with a maximum glucose conversion of 95.76 ± 1.01% (*τ* = 131 min) and NADH productivity of 0.166 ± 0.01 mmol/(L·min) (*τ* = 7.11 min), while maintaining the operational stability over five days. Furthermore, effective diffusion rates comparable to those in aqueous solutions confirmed the suitability of alginate gels for continuous biocatalysis.

In addition, the study investigated the diffusion characteristics of glucose and NADH through the alginate gel and found effective diffusion values similar to those in aqueous solutions, supporting the suitability of alginate for continuous-flow biotransformation.

In summary, this study introduces an innovative and versatile 3D-printed millireactor design that not only addresses the key limitations of the conventional rectangular millireactors, but also decouples the reactor design from the chosen enzyme immobilization method. This flexibility significantly broadens the applicability of the reactor and makes it a universal platform suitable for various biocatalytic processes. The dual-surface immobilization strategy not only proved to be the most efficient for glucose oxidation, but also highlighted the crucial role of maximizing the surface area in improving the catalytic efficiency. This approach opens the door for the development of next-generation bioreactors with tailored enzyme immobilization techniques that can meet specific reaction requirements while maintaining high productivity and stability. Furthermore, this design provides a robust platform for various applications, from biosynthesis to pharmaceutical production and environmental monitoring. Future work could explore scaling up this reactor, testing alternative materials for millireactor fabrication and different materials for enzyme entrapment to further improve the performance and durability, as well as testing other enzymes and reaction systems.

Overall, this work presents an advanced millireactor design that not only improves the reaction efficiency but also provides a robust platform for biotechnological applications with immobilized enzymes.

## Data Availability

The original contributions presented in the study are included in the article, further inquiries can be directed to the corresponding authors.
